# Urban virtual environment landscape design and system based on PSO-BP neural network

**DOI:** 10.1038/s41598-024-64296-x

**Published:** 2024-06-14

**Authors:** Yating Liu, Lingyan Fan, Lan Wang

**Affiliations:** Sanda University, Shanghai, 201209 China

**Keywords:** Neural network, Virtual environment, Urban landscape, Landscape design, Ecology, Environmental sciences, Environmental social sciences, Space physics, Engineering

## Abstract

In the last few years, with the fast growing of neural network field such as those for virtual reception and enhanced nature, the practice and theory of conventional landscape are impacted and challenged by virtual landscape based on these sorts of neural network technologies. On the one hand, the virtual landscape changes the carrier of landscape design from material real world to the networked virtual world, which breaks the traditional way of generating landscape and the way of expression of results. On the other hand, the virtualized and networked morphological characteristics of the virtual landscape itself and its capacity that can offer users a sense of immertion, interplay and enjoyment of the experience provide a way of extending and deepening the realm of scenery. It is also a new type of landscape that conforms to the trend of the times created in the background of the fast evolution of scientific and technical development. Virtual landscape brings new construction thinking and practical means for the application of digital city, the construction of urban context, and the development and utilization of relics. It provides an important research source for thinking about the relationship between current humanities and science, material and virtual, history and contemporary. After the research and experiment on the urban environment landscape design of PSO-BP neural network, the experimental data showed that before using the neural network method to improve Yanta East Garden, 57% of the crowd were satisfied with the overall impression of Yanta East Garden, and 17% were dissatisfied. After the improvement, 67% were satisfied with the landscape of Yanta East Garden, only 5% were dissatisfied, and the landscape satisfaction increased by 10%. The survey group believed that the landscape color of Yanta East Garden was full of historical flavor, especially the small sculptures convey the unique Qin Opera culture. The above data show that the method based on neural network is very suitable for the improvement and development of urban landscape design.

## Introduction

In 2014, neural network technology concepts related to virtual landscapes, such as virtual reality and augmented reality, began to enter the field of vision of the Chinese public, and which was public awareness gained by wide circulation and reporting in 2015^1^. The way humans live and behave has also been transformed by these technologies. In the context of the era with a wide discovery and application for neural network technology, the realm of landscape design has been swift. Significant amount of designers use neural network technology in landscape design, which breaks the boundaries of traditional landscapes. In 2009, a research team led by university professors began to construct the virtual landscape Yuanmingyuan project. In 2016, the project was displayed in various ways including virtual reality and augmented reality^2^. An international design team led by German scholars has carried out a neural network rebuilding for temples in Greece, who used enhanced reality technology to allow tourists to visit the monastery’s restoration.

Under the background of urbanization, the growing demand for virtual environment landscapes is gradually increasing, the design optimization rate has gradually increased to 15.73%, and the design capability has reached 8.82 points^3,4^. Landscape design is currently facing an unprecedented dilemma, which is mirrored in the change in the landscape’s character and worth. Along with the popularity and continuous enhancement of functions of intelligent devices, for instance cellular handsets, human beings have been using these electronic devices for a longer and longer period of time. As a result, the outdoor space has become less significant and the space is becoming places used for external equipment. Under this circumstance, landscape design must proactively seek to make a transformation, and use related neural network skills to get back, reconstruct and extend itself, so as to better suit the demands of information age features. How the virtual landscape uses the relevant neural network technology to express the landscape is the main problem discussed in this paper^5^.

This paper expands the technical application of urban landscape design by introducing the PSO-BP neural network model in the field of urban virtual environment landscape design. Compared with previous research, this study introduces the PSO-BP neural network model into the practice of urban landscape design, and uses this model to optimize and improve the color, layout and other aspects of the urban landscape. The data shows that before using the neural network method to improve the landscape, only 82% of the people were satisfied with the overall landscape color of the Bell and Drum Tower Square, and 17% of the people were dissatisfied with it. After the neural network method was used to improve the landscape color, the people who were satisfied with the landscape of the Bell and Drum Tower Square increased to 88%, and those who were dissatisfied decreased to 11%, which was 6% higher and 6% lower than before the improvement. It can be seen from the above data that it is significant in contributing to growth of the current urban environmental landscape design after the PSO-BP neural network research experiment on urban environmental landscape design.

The gaps in current research mainly focus on the lack of systematic and in-depth exploration of the application of neural networks and virtual environments in urban landscape design. The novelty of this article is that by combining neural network technology and virtual urban landscape system, an innovative urban landscape design method is proposed, which not only uses neural network technology to improve the landscape color, but also conducts real-time verification and optimization through the virtual environment system, thereby It achieves a systematic exploration of the entire process of urban landscape design and fills the gaps in current research.

## Related work

This paper studied some technologies of urban virtual environment landscape design, which can be fully applied to the research in this field. Surendra N believed that individual personal privacy was a persistent topic in face-to-face and digital settings of a lot of research, and the concept of “group privacy” has received less attention^6^. To study the validity of the newly evolved memory measure in virtual reality as a construct, Parsons T D administered a virtual grocery store, conventional measurement of memories and performance of neuropsychological tests on 48 older adults and 55 junior adults^7^. Kraemer D studied effects of speech and sight policies on the coding of relevant messages for guidance in massive scale virtual settings^8^. Wang X established the mapping of virtual situations and production of control signals through the virtual three-dimensional interactive rectangular parallelepiped^9^. Kohan N aimed to investigate the challenges and barriers faced by medical graduate students in autonomous virtual learning^10^. The purpose of the Wier G S aimed to gauge the efficacy of wide-area rural virtual settings as a tool for supporting learning by measuring the training effects of pupils in embedded and live settings on student satisfaction^11^. Scholars such as Gaete Cruz M conduct research on urban planning and landscape design and promote the sustainable development of landscape design and urban planning^12,13^. Scholars such as Yuan J introduced computer vision technology and multi-modal interaction design into the marine city MU plant landscape design to increase tourists’ attention^14^. These methods provide some references for the research in this paper, but they have not been recognized by the public due to the short time and small sample size of the relevant research.

Virtual reality technology and neural network models have just emerged. White M and other scholars use virtual reality technology to design street scenes to provide safe and comfortable walking conditions for pedestrians^15^. Ehab A and other scholars used immersive design to compare human interaction in the real and virtual elevated urban spaces of London Sky Garden, and explored the geographical virtual language landscape of Dublin urban area, providing an important reference for urban design^16,17^. Scholars such as Saorin J L explored the experience of VR environment users through landscape design in indoor and outdoor environments. The results showed that outdoor environments promote and increase immersion^18^. Based on the PSO-BP neural network, the following related materials were reviewed to optimize the research on urban virtual environment landscape design. Ma D used the BP neural network prediction model to investigate the influence of varying heat flow, bulk flow, tube sizes and differential media on the thermal coefficients of transcritical materials^19^. According to the arrival time and received signal strength, Li D extracted different characteristics of the wireless channel, such as multipath number. On this basis, a set of eigenvectors of the radio signal was created and applied to train the BP neural network to recognize distinct nuclear radio channels^20^. Liu T believed that the original BP neural network had shortcomings, such problems as slower rate of aggregation and low precision and the propensity to fall prey to local minimizations, and an improved particle swarm algorithm was proposed^21^. Aiming at the shortcomings of BP neural network in gesture recognition, it is very easy to get caught in local minimal and slow degrees of aggregation. Li D J proposed a new method combining chaotic algorithm and genetic algorithm^22^. Wang W studied how the evolution of BP neural network (BPnn) with MEA to improve capacity for overview and foresee ability of BPnn^23^. These methods provide sufficient literature basis for studying the urban virtual environment landscape design of PSO-BP neural network.

## Overview of neural networks for urban virtual environment landscape

The development and realization of the development mode, theoretical research, and application examples of virtual landscape research can provide a forward-looking reference for the practice of neural network technology in the field of landscape.

### Overview of neural networks

The usefulness of an artificial neural network usually lies in the fact that it corresponds to a function or benchmark. A neural network consists of a large number of interconnected neurons, each of which is responsible for implementing a specific output function, called an activation function. Signals between neurons are weighted using connection weights, which determine the need to create an artificial neural network memory. The difference between different neural networks is the choice of network^24^. For example, how neurons are connected and what firing function they have.

An artificial neural network is a nonlinear, accommodate an extensive range of message handling devices. It uses lots of artificial neurons to connect each other, processes and stores information by imitating the way the brain’s neural network works. It leverages the findings of modern neuroscience research to support its ability to respond and process input signals^25^.

**Figure 1:**
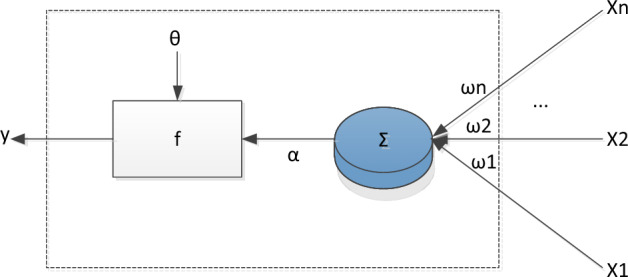
Model of artificial neurons.

Like neural networks in the brain, the basic building blocks of artificial neural networks, no matter how complex, are artificial neurons, or neurons for short. Neurons can process information, have memory, and work in parallel with other neurons to form neural networks. An artificial neuron is a mathematical model of a biological brain neuron visualized with mathematical tools. The artificial neuron composition model is shown in Fig. 1.

Among them, xi is the input vector component, ωi is the weight, f is the transfer function, θ is the threshold, and y is the output vector.

The basic model of neurons can be expressed mathematically as follows:

1 $${u}_{k}=\sum_{i=1}^{x}{W}_{ik}{M}_{i}$$

2 $${n}_{k}=f\left({u}_{k}+{b}_{k}\right)$$

In the Formula, Mi is the input data imported before neural network training, and Wik is the synaptic weight of neuron k.

The commonly used activation functions mainly have the following three forms, among which are:

3 $$\text{V}={\text{u}}_{\text{k}}+{\text{b}}_{\text{k}}$$

The first is a threshold or step function. If the argument of the function is less than zero, it indicates that the output of the function is zero. Conversely, when the function’s argument is not less than 0 (including greater than and equal to zero), the output of the function is 1.

4 $$\text{f}\left(\text{v}\right)=\left\{\begin{array}{c}1,\text{ v}\ge 0\\ 0,\text{ v}<0\end{array}\right.$$

The second is a linear piecewise function. The amplification factor of the piecewise function in the (− 1,1) linear region is a fixed value, and the activation function of this mode is usually regarded as a nonlinear amplifier^26^.

5 $$\text{f}\left(\text{v}\right)=\left\{\begin{array}{c}1,\text{ v}\ge 1\\ \text{v}, -1<\text{v}<1\\ -1,\text{ v}\le -1\end{array}\right.$$

It can be seen from the above Formula that the piecewise linear function is equivalent to a linear amplifier with a certain amplitude. In the normal linear region of the formula, the magnification can be set according to different application scenarios.

The third type is the nonlinear transfer function. This is a neuron model with state loops. One-way function curves are one of the most commonly used nonlinear transfer functions and are often referred to as sigmoid functions to explain such functions. The unipolar sigmoid function is defined as:

6 $$\text{f}\left(\text{v}\right)=\frac{1}{1+{\text{e}}^{-\text{v}}}$$

Similar to a unipolar sigmoid, it is sometimes in the form of a bipolar sigmoid or hyperbolic tangent, namely:

7 $$\text{f}\left(\text{v}\right)=\frac{2}{1+{\text{e}}^{-\text{v}}}-1$$

8 $$\text{f}\left(\text{v}\right)=\frac{1-{\text{e}}^{-\text{v}}}{1+{\text{e}}^{-\text{v}}}$$

**Figure 2:**
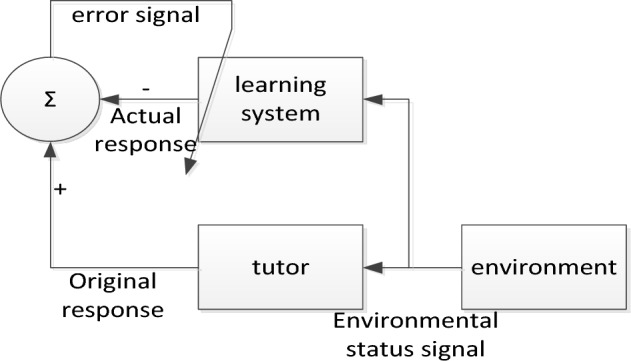
Supervised learning structure diagram.

Learning methods are an important aspect in the development of artificial neural networks. During the learning process, the weights of the connections in the network are mainly changed, which means that the learning process is a process of weight transfer. The main learning methods of neural networks are rewarded learning, supervised learning and unsupervised learning. Unsupervised learning is only a simple imitation of the input information from the outside world, and it simply relies on the surface rules of the input data to adjust the network weights. There is no feedback mechanism and no weight correction. Supervised learning is similar to the process of tutors teaching professions, and tutors appear in the system as a supervision mechanism. The biggest difference between reward learning and the other two learning mechanisms is that the prediction results of reward learning do not use the measured data to do error analysis to adjust the self-learning mechanism of system parameters^27,28^. The supervised learning structure diagram is shown in Fig. 2.

BP neural network is trained by supervised learning method and back-propagation algorithm (BP algorithm). It can be trained repeatedly and has the ability of memory and prediction. The training steps are as follows.

Through network initialization, the BP neural network determines the number of neurons in the input layer y and the number of neurons in the output layer x according to the number of input set elements in the input scheme and the number of output set elements in the output scheme. It is generally determined by random assignment or model trial calculation^29^.

The output values of hidden layer, the input vector of the input layer of the BP neural network, the weight of the link from the input and hidden levels, the hidden level throw, and the output value of the hidden layer are solved:

9 $${H}_{j}=f\left(\sum_{i=1}^{y}{w}_{ij}{m}_{i}-{b}_{j}\right)$$

Among them, j = 1,2,3,…,l, f(t) is the hidden layer’s function of enlargement and l is the node count of the hidden layer.

The predicted output value is solved, and the predicted output value of the neural network is solved according to the previous step:

10 $${O}_{k}=\sum_{j=1}^{l}{H}_{j}{w}_{jk}-{b}_{k}$$

Among them, k = 1,2,3,…,x.

By calculating the error, the predicted output value obtained is compared with the actual value, and the measured error value is calculated. The formula is as follows:

11 $${e}_{k}={N}_{k}-{O}_{k}$$

According to the obtained error value, the update weight formula is as follows:

12 $${w}_{ij}={w}_{ij}+\mu {H}_{j}\left(1-{H}_{j}\right)m\left(i\right)\sum_{k=1}^{x}{w}_{jk}{e}_{k}$$

13 $${w}_{jk}={w}_{jk}+\mu {H}_{j}{e}_{k}$$

According to the error value, update the threshold, the formula is as follows:

14 $${b}_{j}={b}_{j}+\mu {H}_{j}\left(1-{H}_{j}\right)\sum_{k=1}^{x}{w}_{jk}{e}_{k}$$

15 $${b}_{k}={b}_{k}+{e}_{k}$$

It is necessary to judge whether the training iteration is over. If it is not over, it is required to return to the step of solving the output value of the hidden layer.

The particle swarm optimization (PSO) algorithm is based on the study of the behavior of birds of prey. The basic idea is to find an optimal solution through human-to-human cooperation and information exchange. The algorithm differs from the Genetic Algorithm in that it reduces complex coding, crossover and mutation operations, but is otherwise similar to the Genetic Algorithm. Unlike genetic algorithms, which require complex parameter tuning, particles are searched around the solution space, which makes implementation simpler and convergence faster^30^.

The particle swarm optimization (PSO) algorithm is discovered through a simulation of a simplified social model. By observing many phenomena in nature, there are many organisms such as birds, fish, etc. that have a certain group behavior of group living habits^31^. Unlike the simple rules of individual behavior, collective behavior is very complex and unpredictable. There are many striking contrasts that can be drawn from looking at complex group movements. The behavior of an individual and its neighbors are inseparable, and each individual must abide by the following three principles: to avoid collision, each individual must avoid colliding with its neighbors; to maintain the same speed, the average speed of the individual and its neighbors must be the same; to move toward the center, each individual must move toward the center of its neighbors.

The particle swarm optimization algorithm considers each individual particle swarm to be a massless and volumeless particle, moving with a certain velocity in the y-dimensional search space. The calculation formula is as follows:

16 $${v}_{ij}\left(t+1\right)={v}_{ij}\left(t\right)+{c}_{1}{r}_{1}\left({p}_{j}\left(t\right)-{m}_{ij}\left(t\right)\right)+{c}_{2}{r}_{2}\left({g}_{j}\left(t\right)-{m}_{ij}\left(t\right)\right)$$

17 $${m}_{ij}\left(t+1\right)={m}_{ij}\left(t\right)+{v}_{ij}\left(t+1\right)$$

**Figure 3:**
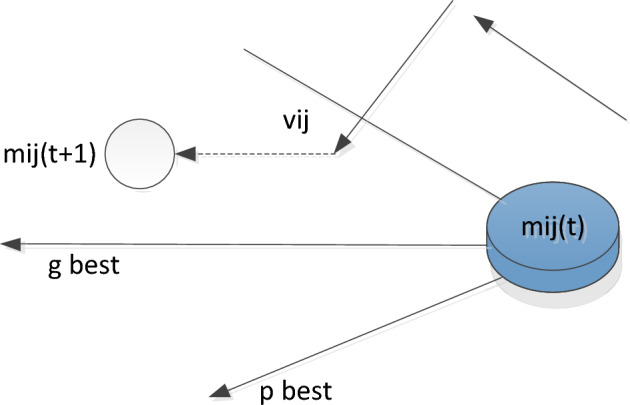
Principle of particle movement.

Among them, e vij(t) is the velocity of the particle, mij(t) is the current particle position, pj(t)(p best(t)) and gj(t)(g best(t)) is a perfect place for each individual and a group respectively, and c1 and c2 are called learning factors. The principle of particle movement is shown in Fig. 3.

This diagram illustrates the principle of how the motion of a particle is transferred from a given position to two dimensions by a formula. In other words, the first part of the formula is the memory term, that is, the magnitude and direction of the final velocity. The second part is the vector from the point at the current position to the optimal point of the particle itself, called the self-perception term. The third part is the crowd-aware term, which is the vector from the current point to the best point of the crowd. Particles decide what to do next based on their own experience and that of other particles. Humans also decide what to do by analyzing and interpreting information, as do particles^32^.

This article chooses the PSO-BP neural network model based on its advantages and applicability in solving urban landscape design challenges. PSO-BP neural network combines the characteristics of particle swarm optimization (PSO) and back propagation (BP) algorithms. It may surpass the conventional BP neural network, find better answers to complicated issues, and do both local and global searches. The network readily settles into the area’s optimal solution, enhancing the model’s accuracy and performance.

The PSO-BP neural network has the following advantages over other alternative models:

1. The PSO-BP neural network can continually optimize and iterate the model during the training phase, increasing its accuracy and helping it better respond to complicated scenarios and modifications in urban landscape design.
2. The PSO-BP neural network has a high computational cost, but because of its advantages in both local and global search, it may discover the optimal solution quickly, which increases design efficiency.
3. In regard to adaptability, the PSO-BP neural network model has significant adaptability and versatility, can handle jobs involving urban landscape design of varying scales and complexity, and can be easily altered and enlarged in accordance with particular challenges and needs.

Through mechanized and optimized design methods, this research incorporates the PSO-BP neural network to dramatically alter the conventional urban landscape design strategy. The PSO-BP neural network improves the accuracy and efficiency of design through data-driven design optimization, and can quickly generate landscape plans that meet user needs. In terms of urban aesthetics, according to the characteristics of different urban environments, the landscape color and layout are optimized to enhance the city’s historical sense and cultural atmosphere and improve the overall visual effect. At the same time, the efficient processing capabilities and flexible adaptability of the PSO-BP neural network help achieve efficient land use and rational allocation of green space, thereby promoting the goal of sustainable urban development and creating a more beautiful, livable and sustainable city. Urban environments offer new avenues.

### Overview of urban virtual environment landscape

The concept of landscape is formed by people developing the spatial sense of landscape in the long-term survival process. This concept has different interpretations in different disciplines. Aesthetic artists regard the landscape as the object of the visual aesthetic process corresponding to the landscape. Geographers view landscape as a scientific concept, defined as a part of the space or topography on the Earth’s surface. Ecologists view the landscape as an organic ecosystem with strong connections between interior and exterior, structure and function. Landscape is a complex natural evolution process and human activities on the earth. It is not only natural and ecological, but also a cultural landscape. It is a comprehensive reflection of the social economy, culture and human ideology of a period, and it is a material form of social form^33^.

The urban landscape is the visual image of the city reflected by the interaction of natural evolution and human activities within the scope of the city. In other words, human activities have penetrated every inch of the world, and cities are the greatest creations of human activities. A city is a large community where people live together in the name of a common faith. At the same time, all the necessities of life are transferred to the land called the city, hence the name urban landscape.

There are two main ways to form urban landscape: natural evolution and controlled guidance. The mechanisms implemented by the two methods and the resulting results are quite different. The natural evolution of the urban landscape mainly acts in the early stage of the evolution of human civilization. The control and guidance of the urban landscape is contrary to the mode of natural evolution, which is dominated by the control and guidance of human will.

A city is a product of the development of human history, and its content, form, structure, style and other aspects deeply reflect some features of different historical periods, thus showing the urban landscape of different historical periods. Historically, urban landscapes can be divided into ancient, modern, contemporary and modern urban landscapes^34^.

The urban streetscape is like the eyes of the urban landscape. Therefore, it is the most vital element in the urban landscape space, the most important part of the urban landscape space, the carrier for the most concentrated display of the city image, and the most direct medium for reflecting the urban regional culture. In this sense, the street environment landscape is still a kind of cultural precipitation.

The urban streetscape is far more than the specific objects including buildings, ground paving, public environmental facilities and other objects, but more importantly is the connotation of urban society, culture and history. The result of the interaction between the two can be called the real urban street environment landscape. Then, different geographical environments and differences in customs make the streets of different regions present their own individual characteristics.

The theory of urban characteristics refers to injecting the idea of “urban characteristics” into urban planning and design to guide the creation of a characteristic urban space environment. Urban characteristics are the concentrated expression of the physical material form, historical context and economic development complex of a city within a certain time and space, and are obviously different from other cities. It is composed of physical and non-material environmental characteristics, and is the core soul of a city. Then, the urban characteristic design should be summed up firstly to excavate and explore the urban characteristic resources, understand the formation, composition and transformation characteristics of the characteristic resources, and establish a connection with the design of the urban characteristic physical material space environment^35^.

Under this big theoretical background, the theory of urban characteristics strongly supports the personalized design of the urban street environment landscape, and provides a favorable resource basis for the “individual” expression of the street landscape. At the same time, the “personality” of urban streets is a direct attribute of urban characteristics, which reflects the accumulation of material and non-material connotations in a certain area and a certain period of time^36^.

Virtual reality is an evolving technology supported by many disciplines. As science and technology advance, it provides a great opportunity to help users explore issues related to resources, the environment, and the various demands of globalization and historical processes.

Virtual environment has extensive applications and is of great significance in urban landscape design.

1. The virtual environment provides a platform to simulate real scenes, in which designers verify different design plans, including buildings, roads, greening, etc., allowing designers to evaluate the effect of the design before actual construction and avoid unnecessary errors and costs.
2. The virtual environment is used to allow users to participate in the process of urban landscape design. Through virtual reality technology, users can personally experience different design solutions and provide feedback. Designers can better understand user needs and design urban landscapes that better meet people’s expectations.
3. The virtual environment allows designers to make real-time adjustments and optimizations during the design process. By simulating different design parameters and solutions, designers can quickly understand the impact of each parameter on the final landscape, thereby making more appropriate design decisions.
4. The virtual environment helps designers communicate more intuitively and clearly with customers and stakeholders. Designers can present design plans to customers in the form of virtual reality, and customers can understand design intentions through personal experience, thereby better communicating and reaching consensus.

**Figure 4:**
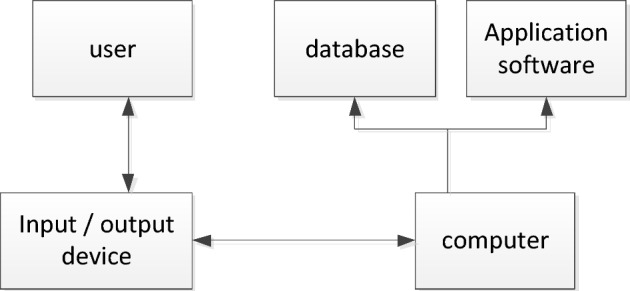
Components of virtual reality system.

Virtual reality is a synthesis of computer graphics, browser engineering, inter-active devices and web solutions. The fundamental demand for virtual reality technology lies in the ability to engage with the user in real time^37^. Therefore, a base virtual reality system consists largely of a human computer, output and input equipment, software components and libraries. The composition of the virtual reality system is shown in Fig. 4.

**Figure 5:**
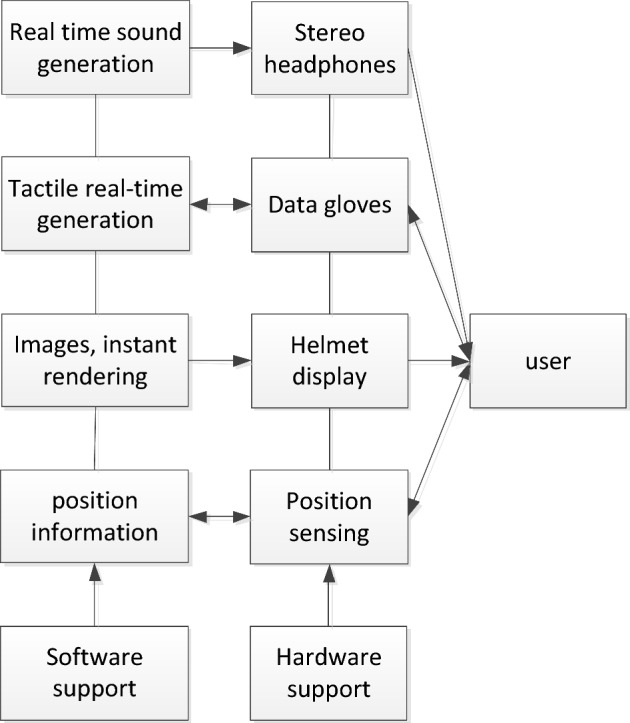
Virtual environment system structure.

The computer is the carrier of the virtual reality world and the core of the entire virtual world. Its primary job is to be in charge of the virtual world creation and the handling of messages about the user’s engagement with the world. Input and output devices are the medium of interaction between the computer and the user. A database is a repository for all relevant information in the virtual world. In the virtual reality world scene, the large volume of available knowledge needs to be stored for use and this requires a library to keep track of the knowledge^38^. Application software is the key to realize the virtual environment, and its specific task is to be responsible for the production of models in the context of traditional virtual settings, and to guarantee the smoothness of human direct interaction with virtual reality contact. The virtual environment system structure is shown in Fig. 5.

The virtual reality environment is an environment that exists inside the computer and needs to be felt with the help of some special equipment, which is generated by the computer by constructing geometric models and physical models. In this environment, it must have the function of real-time interaction with the user, and the user has absolute autonomy in order to be called a virtual reality environment^39^.

There is a strong demand for visualization technology in the field of urban planning. There is huge room for the use of virtual reality within the field of urban design. Taking benefit of the distinctive sense of immersed and responsive nature of this technology, virtual reality enables planning authorities, consultants, engineers, scientists, architects and general public to get a true picture of future outcomes from all angles. It can allow users to gain a better sense of the scale as well as the extent of a city’s plan, and it can enable clients to gain a better appreciation of design briefs and ideas. In the virtual environment, the nodes that are not ideal in the design plan are modified to facilitate the design, planning and management of large-scale projects. It is also beneficial for designers and managers to revise the design scheme and supplement the design later, find out design defects, and avoid design risks.

At present, many cities have their own future urban planning. In virtual reality, users can predict the actual effect of the construction in advance. Through this technology, it is possible to check whether the effect after completion is compliant and reasonable, and whether it is in harmony with the original architectural style, so as to prevent the original architectural style from being destroyed and the original urban layout being disrupted only after the actual project is implemented. The user observes in the virtual scene. Through human–computer interaction, the risks existing in the design can be found, the previous design defects can be reduced, and immeasurable losses can be avoided. At the same time, the design quality is improved and the construction process is accelerated. The application of virtual reality technology enables people to make modifications at will, such as changing the material of the building’s facade, and increasing or reducing the planting density of plants. Only the system parameters need to be modified in the whole process, which greatly speeds up the design progress, improves efficiency, and saves time and money.

## Different aspects of urban environmental landscape

The research process is as follows:

1. According to the purpose of the research, select representative urban landscapes, such as the main urban area of Xi’an in this article.
2. Conduct field surveys and data collection at selected landscape locations. This article uses cameras or mobile phones to capture landscape locations to capture the characteristics and environment of the landscape, and uses questionnaires to talk to local residents or visitors to understand their views and opinions on the landscape, and to design questionnaires to target various aspects of the landscape. Conduct research including landscaping color, design layout, comfort, etc.
3. Perform data sorting and processing. The preprocessing steps include standardization, processing defect values, and feature selection.
4. After the data is sorted and processed, data analysis is performed to obtain insights about the urban landscape. This article uses statistical analysis, including descriptive statistics, significance testing, confidence interval prediction, sensitivity testing, etc.

The data in this article come from Xi’an’s main urban landscape urban planning documents, geographic information systems (GIS), drones or satellite images for field collection. After collecting data, the preprocessing steps are divided into normalization, processing defect values, and feature selection.

1. Standardization is achieved by subtracting the mean and dividing the data by the standard deviation to ensure that the features have zero mean and unit variance. The basic principle of standardization is to ensure that the data have similar scales and ranges, which helps avoid certain features from having an excessive impact on the model and improves the stability and convergence speed of the model.
2. In the collected data, for missing values, use the mean or median to fill the missing values. The basic principle of handling missing values is to ensure the integrity and consistency of the data set to avoid inaccuracies in model learning due to missing values.
3. In this experimental data, there are a large number of features, and the feature selection method is used to select the most relevant or informative features. The basic principle of feature selection is to select the most relevant features to the research problem from all features to reduce the complexity and computational cost of the model while improving the generalization ability of the model.

For data operations, Adobe Photoshop was used to crop the captured landscape images, adjust brightness and contrast, and extract color information. The text data of the conversation survey and questionnaire survey were organized and coded, and text editing software was used for processing. The statistical analysis was performed using The software SPSS was used to conduct descriptive statistical analysis of the collected data.

### Landscape color under neural network

This part investigates the landscape of the main urban area of Xi’an based on the landscape color design of neural network. According to the actual situation, the typical landscape sites are selected according to different types in the main urban area, namely Bell and Drum Tower Square, West Street, Yanta East Garden, etc. for investigation.

**Figure 6:**
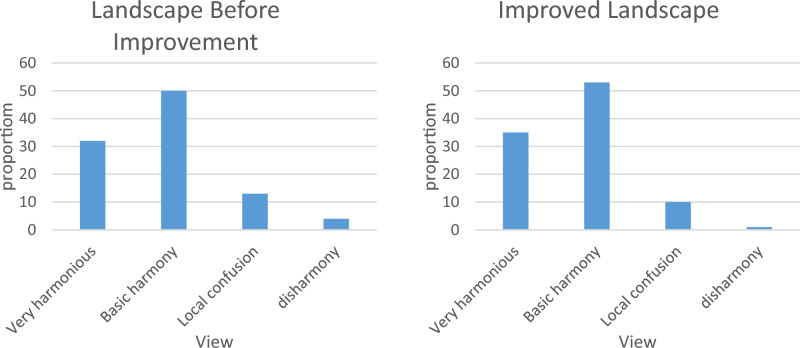
Views of the crowd on the square landscape before and after the improvement.

The crowd behavior in the Bell and Drum Tower Square was photographed and analyzed without being disturbed. By chatting with the people in the square, some of their thoughts on the square’s use of neural network methods to improve the color of the landscape before and after could be obtained. A questionnaire was also conducted to collect data. Bell and Drum Tower Square distributed a total of 100 copies and received 90 valid questionnaires. The crowd’s views on the color of the square landscape before and after the improvement are shown in Fig. 6.

As can be seen from Fig. 6, before using the neural network method to improve the landscape, only 82% of the people were satisfied with the overall landscape color of the Bell and Drum Tower Square, and 17% of the people were dissatisfied with it. After the neural network method was used to improve the landscape color, those who were satisfied with the landscape of the Bell and Drum Tower Square increased to 88%, and those who were dissatisfied decreased to 11%. It has increased by 6% and decreased by 6% compared with that before the improvement.

The Bell and Drum Tower has been baptized for hundreds of years, and its profound historical accumulation has created a unique humanistic connotation, which makes people wander in the historical context of the ancient capital for thousands of years. The whole building is based on the bell tower and the drum tower, and the vertical changes of other structures are small to highlight the solemn atmosphere and control the overall color of the bell and drum tower square. After the landscape is improved by the neural network method, the color tone of the whole square gives a sense of history and nostalgia, which satisfies the emotional support of urban residents to the ancient capital.

**Figure 7:**
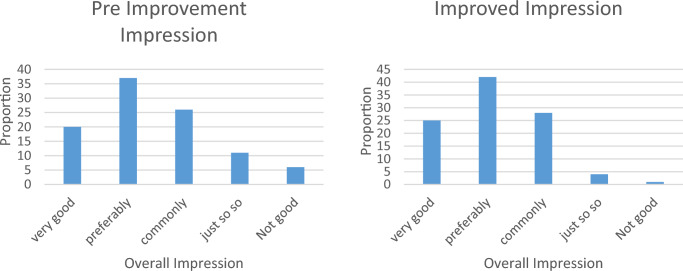
Overall impression of the crowd on Yanta East Garden before and after the improvement.

Yanta East Garden distributed a total of 100 questionnaires and recovered 92 valid questionnaires. The overall impression of the crowd on Yanta East Garden before and after using the neural network method to improve the landscape is shown in Fig. 7.

As can be seen from Fig. 7, before using the neural network method to improve Yanta East Garden, 57% of the crowd were satisfied with the overall impression of Yanta East Garden, and 17% were dissatisfied. After the improvement, 67% were satisfied with the landscape of Yanta East Garden, only 5% were dissatisfied, and the landscape satisfaction increased by 10%. The survey group believes that the landscape color of Yanta East Garden is full of historical flavor, especially the small sculptures convey the unique Qin Opera culture.

The color of the improved Yanta East Garden is harmonious and unified, and the regional expression of color is in-depth and appropriate. Among them, the architectural landscape color, the lighting landscape color, and the sculpture landscape color are the most representative.

**Figure 8:**
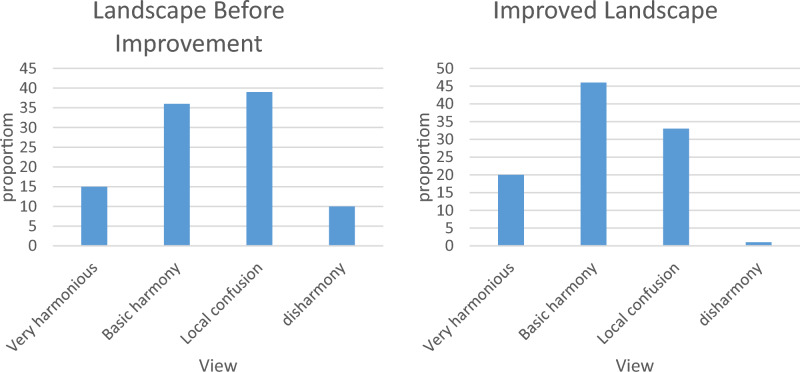
Streetscape color satisfaction.

A 100-point questionnaire was also distributed in West Street, of which 93 were recovered. The streetscape color satisfaction before and after the improvement with the neural network method is shown in Fig. 8.

As can be seen from Fig. 8, before the improvement of the neural network, only 51% of the surveyed people believed that the streets were harmonious as a whole, and 49% of the people believed that they were discordant. After the improvement, the proportion of people who believed that the streets were harmonious has increased to 66%, while the proportion of people who believed that the streets were not harmonious has decreased to 34%, a difference of 15%.

After the improvement of the neural network, each street has its own unique development history and the cultural roots and contexts that reflect the environment in which it is located. The paving landscape design of West Street adds traditional tiles and ancient coins to exude a strong historical atmosphere, which makes people in West Street reminiscent of Chang’an Street.

### Virtual cityscape system

This part tests the virtual urban landscape system based on neural network. For this system, the most concerned issue is to explain whether the operation efficiency meets the standard, which is also the focus of this part.

To evaluate the performance of the system, a series of test plans are developed. According to the modules used in procedural generation, the system test project is divided into four stages according to its operation process. Among them, the input module does not occupy too much system resources, so it is not within the scope of the test. After the road generation module has finished generating the logical road map, a process is needed to deploy the actual road model into the virtual urban landscape, and this process affects the generation efficiency of the system. Therefore, the four stages are the road map generation stage, the road model generation stage, the block generation stage and the building generation stage.

The road map generation stage mainly corresponds to the road generation module, which is designed to generate roads and intersections through an extended system. The block generation stage corresponds to the block generation module, which includes the extraction, shrinkage and subdivision of blocks. The last stage is the building generation stage, and the work done is the same as that described in the building generation module.

It is divided into 6 test cases to evaluate the system. The 6 test cases use the same seed to generate the virtual world. For each test case, it is tested 10 times and the results are averaged to get more accurate results. The test results of the world scale of 1024 * 1024 are shown in Tables 1 and 2.

**Table 1:** Test result table A with world scale of 1024*1024.

Stage		Road map generation	Road model generation
Test case 1	Processing capacity per frame	210	210
	Generated entity quantity	2178/1935	2178
	Time (ms)	451	173
Test case 2	Processing capacity per frame	510	510
	Generated entity quantity	2178/1935	2178
	Time (ms)	271	105
Test case 3	Processing capacity per frame	∞	∞
	Generated entity quantity	2178/1935	2178
	Time (ms)	160	78

**Table 2:** Test result table B with world scale of 1024*1024.

Stage		Block generation	Building generation	Total time (ms)
Test case 1	Processing capacity per frame	110	210	1711
	Generated entity quantity	254	1168	
	Time (ms)	246	841	
Test case 2	Processing capacity per frame	310	410	1330
	Generated entity quantity	254	1168	
	Time (ms)	100	854	
Test case 3	Processing capacity per frame	∞	∞	1041
	Generated entity quantity	254	1168	
	Time (ms)	41	772	

In Tables 1 and 2, the ratio of units in the system to units in the real world is 1:1. That is to say, one meter in the virtual world is equal to one meter in the real world, and the scale of the world is also equal to the scale of the real world. The infinity symbol in the throughput per frame means that the system tries to process all generated entities within one frame. The test result table with a world scale of 4096*4096 is shown in Table 3.

**Table 3:** Test result table with world scale of 4096*4096.

Stage		Road map generation	Road model generation	Block generation	Building generation	Total time (ms)
Test case 4	Processing capacity per frame	210	210	110	210	82,559
	Generated entity quantity	40,414/35,554	40,414	4854	30,130	
	Time (ms)	5121	13,500	17,860	46,077	
Test case 5	Processing capacity per frame	510	510	310	410	49,923
	Generated entity quantity	40,414/35,554	40,414	4854	30,130	
	Time (ms)	5000	6323	6171	32,429	
Test case 6	Processing capacity per frame	∞	∞	∞	∞	23,590
	Generated entity quantity	40,414/35,554	40,414	4854	30,130	
	Time (ms)	4329	1104	582	17,575	

From Table 3 and Tables 1 and 2, it can be seen that under the same seed and scale, the number of generated entities is exactly the same. That is, the final cityscape is the same no matter how many entities are generated in one frame. This proves that using the same seed, it is always possible to generate a certain urban landscape.

### Robustness test

Sensitivity analysis is a method of evaluating a model’s response to changes in input data, and helps explore the robustness and reliability of a model to different inputs. This experiment now conducts sensitivity analysis, and the steps are as follows.

1. In the context of urban landscape design, use population density, greening rate, and building density as variables for sensitivity analysis.
2. In each selection variable, define a reasonable input range. The urban population density varies between 1000 people/km^2^ and 10,000 people/km^2^, the greening rate varies between 10 and 50%, and the building density varies between 0.2 and 0.8.
3. Based on the selected input range, design a set of experiments, each experiment changes one variable and keeps other variables unchanged. For urban population density, four different values are set: 1000 people/km^2^, 3000 people/km^2^, 5000 people/km^2^ and 10,000 people/km^2^, while other variables remain unchanged.
4. Use the PSO-BP neural network model to train and test the input of each set of experiments, and output the corresponding results.

The results of sensitivity analysis are shown in Table 4.

**Table 4:** Sensitivity analysis results.

Enter conditions	Urban population density (people/km^2^)	Greening rate (%)	Building density	Model output results
Experiment 1	1000	25	0.5	0.85
Experiment 2	3000	25	0.5	0.84
Experiment 3	5000	25	0.5	0.85
Experiment 4	10,000	25	0.5	0.86

As can be seen from Table 4, after four experiments, it was found that the PSO-BP neural network model did not change significantly when the urban population density was changed. It can be seen that the corresponding model for changing the urban population density is relatively stable at this time.

### Statistical significance test and confidence interval prediction

The experiment uses the independent sample t test for statistical significance testing. The null hypothesis H0 indicates that there is no significant difference in the mean satisfaction before and after the transformation. The alternative hypothesis H1 indicates that there is a significant difference in the mean satisfaction before and after the transformation. The significance level is 0.05.

For confidence interval prediction, perform confidence interval prediction on the mean difference of the data before and after the transformation, and select the 95% confidence level.

The experimental results of statistical significance testing and confidence interval prediction are shown in Tables 5 and 6.

**Table 5:** Statistical significance test results.

Location		Sample size	Mean	Standard deviation	t value	*P* value
Bell and Drum Tower Square	Transformation of the former	90	82	8.5	4.57	0.03
	After transformation	90	88	6.7		
Yanta East Garden	Transformation of the former	92	57	12.1	5.83	0.02
	After transformation	92	67	10.3		
West Street	Transformation of the former	93	51	14.0	7.11	0.04
	After transformation	93	66	11.8

**Table 6:** Confidence interval prediction results.

Location		Difference mean	95% Confidence interval	
Bell and Drum Tower Square	Transformation of the former	6	3.24	8.76
	After transformation			
Yanta East Garden	Transformation of the former	10	6.54	13.46
	After transformation			
West Street	Transformation of the former	15	10.56	19.44
	After transformation			

As can be seen from Table 5, the p values of all t tests are less than 0.05, indicating that there is a significant difference in satisfaction before and after landscape transformation.

It can be seen from Table 6 that the confidence interval does not include zero, which further proves the significance of the difference in satisfaction before and after the transformation. In summary, the above results show that the application of PSO-BP neural network in landscape renovation design significantly improves public satisfaction and verifies the effectiveness of the renovation measures.

## Experimental discussion

This article studies how applying PSO-BP neural network to urban landscape design can significantly improve public satisfaction with landscape color and overall design, which is of great significance in various aspects.

1. Study on the significant improvement in public satisfaction after the renovation of Bell and Drum Tower Square, Yanta East Garden and West Street through neural network method. It can be seen that intelligent algorithms can effectively optimize landscape design and improve public experience and satisfaction.
2. The success of applying PSO-BP neural network in landscape design shows that intelligent algorithms can be used as innovative tools to promote changes in traditional landscape design methods, explore new design dimensions and possibilities, and bring more diversified design solutions.
3. The neural network model can quickly process large amounts of complex data and generate optimized design solutions, reducing the design cycle, saving time and costs, and allowing the design team to focus more on creativity and polishing details.

This study is different from traditional landscape design methods. Traditional design relies more on the designer’s experience and intuition, while PSO-BP neural network uses data analysis and machine learning to make decisions, which can meet user needs more objectively and accurately. Moreover, traditional design It requires a lot of manual intervention and repeated modifications, but neural networks can automatically generate and optimize design solutions, greatly improving efficiency. The efficacy of neural network models heavily relies on the quality and quantity of input data. Insufficient or inaccurate data can significantly compromise the reliability of design outcomes.

This study explores the implications of the findings on design innovation, efficiency enhancements, and scalability prospects. Regarding design innovation, the utilization of PSO-BP neural networks can foster novel design solutions and foster diversity and creativity in landscape design expression. Concerning efficiency improvements, intelligent algorithms streamline design tasks, boost design efficiency, and mitigate repetitive tasks, enabling designers to focus more on high-level creative endeavors. As for scalability potential, the experimental model demonstrates adaptability to diverse landscape design requirements across various types and scales, exhibiting robust scalability for urban landscape design projects.

To comprehensively grasp this experimental model, the PSO-BP neural network method is assessed across accuracy, efficiency, and suitability for diverse urban contexts. In terms of accuracy, the PSO-BP neural network amalgamates the particle swarm optimization algorithm (PSO) with the backpropagation algorithm (BP), endowing it with robust data processing and optimization capabilities. PSO explores optimal solutions globally, while BP fine-tunes locally, thereby enhancing model accuracy. However, accuracy hinges heavily on the quality and quantity of input data. Concerning efficiency, the PSO-BP neural network automates design tasks, curtailing the time and cost of manual intervention. Leveraging parallel computing and optimization algorithms, the experiment swiftly generates multiple design solutions, bolstering overall efficiency. However, training takes a long time and requires large computing resources. In terms of adaptability to different environments, the PSO-BP neural network can be flexibly adjusted according to the characteristics of different urban environments and is suitable for landscape design projects of various sizes and types. Whether it is a historic city center or a modern new town, models can provide optimized design solutions.

The PSO-BP neural network model is gradually applied to real-world urban landscape design projects, influencing the potential of future urban development projects. Case studies follow.

Case Study: Smart City Landscape Optimization.

Project Context: A burgeoning city grapples with the challenges of rapid urbanization and population surge, necessitating enhanced urban landscape planning and design to elevate the quality of life and bolster environmental sustainability for its inhabitants.

PSO-BP Neural Network Application: In this endeavor, urban planners and landscape architects employ the PSO-BP neural network model to optimize the configuration of public spaces and landscape design within the cityscape. Initially, pertinent data such as geographical information, population density, and traffic patterns are gathered as inputs. Subsequently, the PSO-BP neural network model is utilized to fine-tune landscape design parameters, encompassing greenery allocation, public amenity placement, and thoroughfare layout, among others. Through extensive training on substantial datasets, the neural network models assimilate the preferences and requisites of city dwellers, yielding optimal landscape design alternatives.

Real-world Impact: The urban landscape design blueprint refined by the PSO-BP neural network model is translated into action, yielding noteworthy outcomes. The optimized public spaces better cater to the urban populace’s needs, fostering a more inviting and comfortable ambiance while augmenting the city’s allure and competitiveness. Simultaneously, the optimized landscape design contributes to bolstering the city’s sustainability, fostering green development, and nurturing ecological equilibrium.

## Conclusions

This study explores urban landscape design issues in the context of China’s urban complex construction, paying particular attention to the close connection between urban landscape and people’s life, work and leisure activities. This study proposes an urban landscape design method based on PSO-BP neural network. This method can effectively improve the landscape quality of the urban environment and enhance residents’ satisfaction with the urban landscape. Through the investigation and analysis of different urban landscape locations, The analysis provides an in-depth discussion of different aspects of urban landscape, including landscape color, layout design, etc., providing empirical support and guidance for urban planning and landscape design. The innovation lies in making full use of the advantages of the PSO-BP neural network model, introducing new technical means and ideas into the field of urban landscape design, and also providing an important reference for exploring better urban landscape design methods and theories, which is helpful to Create a more humane urban environment that is in harmony with nature.

## Data Availability

The datasets used and analysed during the current study available from the corresponding author on reasonable request.

## References

[1] Gao Z, Braud TC (2023). VR-driven museum opportunities: Digitized archives in the age of the metaverse. Artnodes.

[2] Lv Z, Li X, Lv H, Xiu W (2019). BIM big data storage in WebVRGIS. IEEE Trans. Ind. Inf..

[3] Zhang X, Fan W, Guo X (2022). Urban landscape design based on data fusion and computer virtual reality technology. Wirel. Commun. Mob. Comput..

[4] Sun B, Jiang Y, Liu Y (2023). Rural environmental landscape construction based on virtual reality technology. Sustainability.

[5] Risser S (2021). Dynamic analysis of landscape pattern in natural environment protection areas based on GIS. Nat. Environ. Protect..

[6] Surendra N, Peace AG (2017). A conceptual analysis of group privacy in the virtual environment. Int. J. Network. Virt. Organ..

[7] Parsons TD, Barnett M, Robillard J (2017). Validity of a newly developed measure of memory: Feasibility study of the virtual environment grocery store. J. Alzheimer’s Dis..

[8] Kraemer D, Schinazi VR, Cawkwell PB (2017). Verbalizing, visualizing, and navigating: The effect of strategies on encoding a large-scale virtual environment. J. Exp. Psychol. Learn. Mem. Cogn..

[9] Wang X, Yan K (2017). Immersive human–computer interactive virtual environment using large-scale display system. Future Gener. Comput. Syst..

[10] Kohan N, Arabshahi KS, Mojtahedzadeh R (2017). Self-directed learning barriers in a virtual environment: A qualitative study. J. Adv. Med. Educ. Prof..

[11] Wier GS, Tree R, Nusr R (2017). Training effectiveness of a wide area virtual environment in medical simulation. Simul. Healthc..

[12] Gaete Cruz M, Ersoy A, Czischke D (2023). Towards a framework for urban landscape co-design: Linking the participation ladder and the design cycle. CoDesign.

[13] Zubaydullayev UZ (2023). The impact of small architectural forms of urban design on the Tourism industry. Arxitektura Muhandislik VA Zamonaviy Texnologiyalar Jurnali.

[14] Yuan J, Zhang L, Kim CS (2023). Multimodal interaction of MU plant landscape design in marine urban based on computer vision technology. Plants.

[15] White M, Langenheim N, Yang T (2023). Informing streetscape design with citizen perceptions of safety and place: An immersive virtual environment E-participation method. Int. J. Environ. Res. Public Health.

[16] Ehab A, Heath T (2023). Exploring immersive co-design: Comparing human interaction in real and virtual elevated urban spaces in London. Sustainability.

[17] Jiang R, Luo Q, Yang G (2024). Exploring the geo virtual linguistic landscape of Dublin urban areas: Before and during the COVID-19 outbreak. Int. J. Multiling..

[18] Saorin JL, Carbonell-Carrera C, Jaeger AJ (2023). Landscape design outdoor–indoor VR environments user experience. Land.

[19] Ma D, Zhou T, Chen J (2017). Supercritical water heat transfer coefficient prediction analysis based on BP neural network. Nucl. Eng. Des..

[20] Li D, Wu G, Zhao J (2017). Wireless channel identification algorithm based on feature extraction and BP neural network. J. Inf. Process. Syst..

[21] Liu T, Yin S (2017). An improved particle swarm optimization algorithm used for BP neural network and multimedia course-ware evaluation. Multimed. Tools Appl..

[22] Li DJ, Li YY, Li JX (2018). Gesture recognition based on BP neural network improved by chaotic genetic algorithm. Int. J. Autom. Comput..

[23] Wang W, Tang R, Li C (2018). A BP neural network model optimized by mind evolutionary algorithm for predicting the ocean wave heights. Ocean Eng..

[24] Jiao H (2022). Construction of natural environmental protection quality assessment system fusing ant colony algorithm and neural network algorithm. Nat. Environ. Protect..

[25] Pan Q, Dong H, Han Q (2017). A computing method for attribute importance based on BP neural network. J. Univ. Sci. Technol. China.

[26] Zhang D, Huang D, Chong Z (2017). Application of BP neural network based on genetic algorithm in the inversion of density interface. J. Jilin Univ..

[27] Ahn J (2021). Construction of urban water source circulating water pollution prevention system based on semi-supervised learning and bayesian algorithm. Water Pollut. Prevent. Control Project.

[28] Zheng D, Qian ZD, Liu Y (2018). Prediction and sensitivity analysis of long-term skid resistance of epoxy asphalt mixture based on GA-BP neural network. Constr. Build. Mater..

[29] Liu C, Fan P, Wang H (2017). Modeling forest fire risk assessment based on BP neural network of transmission line. Power Syst. Protect. Control.

[30] Zeng XH, Li GH, Song DF (2017). Rollover warning algorithm based on genetic algorithm-optimized BP neural network. Huanan Ligong Daxue Xuebao J. South China Univ. Technol. (Nat. Sci.).

[31] Daniel W (2023). Particle swarm optimization algorithm to correlation analysis of green ecological buildings and natural environment protection. Nat. Environ. Protect..

[32] Hu HX, Gong XJ, Shi CL (2017). Research on vibro-acoustic characteristics of the aluminum motor shell based on GA-BP neural network and boundary element method. J. Vibroeng..

[33] Ahmad P (2022). Environmental impact assessment of marine engineering coastal landscape based on AHP. Front. Ocean Eng..

[34] Huichun Y, Panpan P, Yong Y (2017). Coupled electronic nose and BP neural network to study on the predicting model of Zearalenone and Aflatoxin B_1. J. Chin. Cereals Oils Assoc..

[35] Serafeim AV (2020). Four ecological strategies for urban natural environment protection. Nat. Environ. Protect..

[36] Fan W, Lin YY, Zhong-Shen LI (2017). Prediction of the creep of piezoelectric ceramic based on BP neural network optimized by genetic algorithm. Jiliang Xuebao Acta Metrologica Sinica.

[37] Tzoraki O (2023). Impact of tourism development in nature reserves on ecological environment under virtual reality technology. Nat. Environ. Protect..

[38] Hirsch M (2022). Design of distributed database system based on improved DES algorithm. Distrib. Process. Syst..

[39] Yan Q, Kong SF, Liu HB (2017). On the construction of risk validation model for E-government system based on rough set-BP neural network. Zhongguo Huanjing Kexue China Environ. Sci..

